# Musical Expertise and Executive Functions in Experienced Musicians

**DOI:** 10.3390/brainsci13060908

**Published:** 2023-06-04

**Authors:** Edoardo Passarotto, Bruno Kopp, André Lee, Eckart Altenmüller

**Affiliations:** 1 Institute of Music Physiology and Musicians’ Medicine, University of Music, Drama and Media Hannover, 30175 Hanover, Germanyeckart.altenmueller@hmtm-hannover.de (E.A.); 2 Department of Neurology, Hannover Medical School, 30625 Hannover, Germany; 3 Department of Neurology, Klinikum Rechts der Isar, Technical University of Munich; 80333 Munich, Germany

**Keywords:** practice, expertise, executive functions, advanced musicians, cognitive development

## Abstract

Extensive music practice has been suggested to enhance the development of cognitive abilities over and above musical expertise. Executive functions (EFs) have been particularly investigated, given their generalizability across different domains and their crucial role in almost all aspects of cognition. However, the relationship between musical expertise and EFs is still not completely understood, as several studies have reported conflicting results. The present study aims to investigate the relationship between musical expertise and EFs, determining which facets—if any—of EFs might be particularly relevant to extensive music practice. Thirty-five student pianists completed a set of neuropsychological tasks which assessed EFs (the Trail Making Task, Design Fluency, Numerical Stroop, and the Tower of London). They also performed a short musical excerpt inspired by the piano literature. Musical expertise was assessed by considering three parameters, namely the highest academic degree in music, the lifetime amount of music practice, and the quality of the sample-based musical performance. The results indicate that postgraduate piano students did not show advantages in EFs compared to undergraduate piano students. More extensive lifetime practice in music was solely associated with faster visual reaction times on the Numerical Stroop task. The Trail Making and Design Fluency scores were significant predictors of the quality of the sample-based musical performance. In conclusion, the present data suggests that EFs and the amount of music practice do not seem to be correlated in student pianists. Nevertheless, some facets of EFs and the quality of musical performance may share substantial amounts of variance.

## 1. Introduction

Music practice relies heavily on motor and cognitive abilities that are also beneficial to other everyday activities [1,2]. For instance, musicians seem to have superior fine motor control, bimanual coordination, auditory discrimination abilities and musical sophistication, compared to non-musicians [3,4,5,6]. These advantages are likely due to similarities between the behaviors tested in the laboratory and the skills trained in the practice rooms; they are usually considered near-transfer effects. Far transfer instead occurs when music training enhances performance in domains and activities which are not closely related to music, such as mathematical skills [7] and learning a second language [8]. Nevertheless, recent meta-analyses have questioned the reliability of far-transfer effects [9,10].

### 1.1. Executive Functions

Far-transfer effects are likely promoted by domain-general cognitive processes, which may be exploited and trained when practicing music. Executive functions (EFs) have been heavily investigated, given their generalizability across different cognitive domains and their widespread utility in everyday life [11]. 

EFs are a set of abilities which play a crucial role in almost all aspects of cognition. They are usually grouped under three core components: cognitive inhibition, working memory, and set-shifting [12]. In addition, these components form the basis for higher-order EFs such as planning, problem solving and cognitive fluency [13,14]. They develop during childhood and they reach a period of relative stability in early adulthood before they decline in older age [15]. In fact, age-related decline in EFs is already evident in the third and fourth decades of life [16,17,18]. 

Previous studies have shown that musicians perform better than non-musicians in neuropsychological tests measuring working memory [19], set-shifting [20], cognitive fluency [11] and cognitive inhibition [21]. However, these findings are rather inconsistent and several studies have reported conflicting results [13,22,23].

### 1.2. Causal Relationship

Therefore, the causal relationship between music training and EFs is still not completely understood. According to Okada and Slevc [24], their relationship can be represented by three alternative models. First, music training might enhance EFs by drawing on common cognitive resources. Alternatively, optimal EFs might be necessary for skilled music making, and thus musical expertise might be the result of a selection bias: only individuals who possess sufficient cognitive resources can successfully pursue music studies and careers as professional musicians. Finally, EFs and musical expertise might mutually influence each other. Therefore, pre-existing cognitive advantages may facilitate music making, and these individuals may further increase their advantage by training EFs through music practice.

### 1.3. Music Interventions

Causality is usually investigated through longitudinal study designs and music interventions in music-naïve individuals. These studies are usually focused on samples of children and older people. A recent meta-analysis [13] suggests that improvements in cognitive inhibition are the most common outcome of music making in children, while results related to working memory, set-shifting, planning and cognitive fluency are rather inconsistent. Similarly to children, older people seem to benefit from music lessons by improving their gross- and fine-motor coordination [25,26], processing speed and working memory [27], as well as speech perception [28].

Nevertheless, it is not clear which executive functions are specifically improved through music interventions. The findings are inconsistent, probably due to the variety of the experimental designs implemented [13]. Likewise, specific musical activities, which compose musicians’ professional routines, might affect distinct cognitive processes. Thus, knowledge about the cognitive resources that are recruited while making music might help better understand the relationships between musical expertise and EFs. 

### 1.4. Experienced Musicians

Only a few studies have investigated the relationship between musical expertise and EFs in samples of experienced musicians. Slevc and colleagues [29] and Okada and Slevc [24] measured cognitive inhibition, working memory, and set-shifting abilities in both auditory and visual domains in a large sample of university-aged students. Notably, instead of dichotomizing participants into groups, i.e., musicians and non-musicians, they assessed musical expertise by implementing self-report measures of musicality as well as by melody and rhythmic discrimination tests. The results indicated a positive association between musical abilities, expertise and working memory. Set-shifting and cognitive inhibition were unrelated to musical abilities. However, the authors implemented measures of musical expertise that did not consider participants’ proficiency with the instrument, nor other fundamental aspects of musical expertise. In fact, according to a comprehensive review by Mishra [30], musical expertise is a complex construct, which can be assessed by considering three main parameters: academic degrees in music, the amount of practice achieved during lifetime, and the quality of musical performance. None of these parameters was measured in the mentioned studies.

### 1.5. Aim

The present project aims to investigate the relationship between executive functions, musical expertise, and musical performance in a sample of university-aged student pianists practicing a short musical excerpt. Musical expertise was assessed by considering multiple parameters, including performance quality. The results will allow us to determine which facets of EFs might be particularly relevant to music practice and performance. Given the lack of consistency in previous findings, we hypothesize a low level of generalizability of our results across measures of musical expertise.

## 2. Methods

### 2.1. Design

Experienced pianists completed a battery of neuropsychological tasks (NT), and practiced and performed a short musical excerpt. The experimental procedure and the results discussed here are part of a broader research project whose findings have been partially reported in Passarotto and colleagues [31].

### 2.2. Participants

Thirty-five healthy, right-handed pianists took part in the experiment. A total of 60% were females and 40% were males. Their mean age was 24.2 years (*SD* = 3.8, range 18–35 years). All participants were enrolled in music universities in northern Germany as undergraduate (*N* = 15) or postgraduate (*N* = 20) students, majoring in piano and classical music. Only 30 participants were considered for the analyses involving music *performance quality* scores: in fact, 5 participants were excluded, due to their previous experience with the musical task used for the experiment (*N* = 3) or for not following the guidelines given by the principal investigator (*N* = 2). Participation was voluntary, and it was compensated with EUR 50. Written informed consent was obtained from all participants.

### 2.3. Materials

The experiment comprised a battery of neuropsychological tasks, as well as three measures of musical expertise suggested by the literature [30]. Detailed descriptive statistics and operational definitions of individual NT parameters are reported in Table 1.

#### 2.3.1. Design Fluency (DF)

Design Fluency (DF) is a neuropsychological task aimed at assessing cognitive fluency in generating visual patterns. The task was presented in its computerized version developed by Woods and colleagues [18]. During the task, five fixed points were displayed on the screen, and participants were asked to connect them by drawing designs of four continuous lines. The aim of the task was to draw as many unique designs as possible in 90 s, avoiding repetitions. Five supervised practice trials were provided at the beginning of the task. 

#### 2.3.2. Numerical Stroop (nSTROOP)

The numerical Stroop task aims to measure processing speed and response inhibition when evaluating visual stimuli. The nSTROOP task used in the present study was programmed according to the indications set out by Heine and colleagues [32]. However, the number of trials was halved, to reduce completion time to approximately 10 min. The task was divided into two parts, the numerical-magnitude and the physical-size comparison. In each part, participants were asked to decide which of the two numbers shown on the screen was either numerically or physically larger than the other. Participants were asked to answer as quickly and as accurately as possible by pressing two dedicated keyboard keys (Q and P, controlled by the index fingers of the right and the left hand, respectively), indicating either the stimulus on the right or on the left side of the screen. Stimuli were manipulated in terms of physical size and numerical magnitude. They were presented in three different conditions: congruent (i.e., greater magnitude corresponded to bigger physical size, 9 2), incongruent (i.e., smaller stimuli indicated a greater numerical value, 9 2), and neutral, where only one aspect of the stimuli was altered (i.e., only numerical magnitude was altered, 9 2). The task consisted of 96 trials evenly split into parts, that is, the numerical-magnitude and physical-size comparison (48 trials each), and the conditions, namely congruent, incongruent, and neutral (16 trials each). The order of the experimental phases and trials within each phase was randomized across participants. Each part of the task was introduced by ten supervised practice trials. For further details, see Heine and colleagues [32].

#### 2.3.3. Trail Making Task (TMT)

The Trail Making Task measures visual attention and set-shifting. The present study used a computerized version of the task developed by Woods and colleagues [17]. TMT is divided into two conditions, Trail A and Trail B. In the first condition, 25 circles containing numbers ranging between 1 and 25 were displayed on the screen and participants were asked to click them in chronological order as fast as possible, avoiding errors. During Trail B, the 25 circles contained both numbers, ranging between 1 and 13, and letters, from “A” to “L”. Participants were asked to quickly click on the circles, following both numerical and alphabetical order and alternating between the two processes (i.e., 1–A–2–B–3–C). Both trails were preceded by five supervised practice trials. 

#### 2.3.4. Tower of London (TOL)

The Tower of London (Drexel University) (TOL) task is designed to assess planning and problem solving abilities [33]. The task was presented in its computerized version, programmed by Mueller and Piper [34]. During the task, two structures consisting of three columns of different length were displayed on the screen. Each structure contained three disks of different colors (blue, red, and green), with a different arrangement of the disks between the two structures. Participants were allowed to move the disks in only one of them, with the objective of matching the arrangement of the disks between the two structures by using the least number of moves. Moreover, participants were only allowed to move one disk at a time, and they could not pile up more disks in a column than it could accommodate. The task consisted of 15 subtasks of increasing difficulty. For a detailed description, see Culbertson and Zillmer [33].

#### 2.3.5. Musical Expertise

Musical expertise was assessed based on three different parameters suggested in Mishra’s review [30]: academic degrees in music, lifetime practice and performance quality. In the first case, participants were divided into two groups: undergraduate (*N* = 15) and postgraduate (*N* = 20) pianists. *Lifetime practice*, i.e., the cumulative amount of practice during a lifetime measured in hours, was assessed through a dedicated scale developed by Butkovic and colleagues [35]. *Performance quality* was measured through the task and computerized assessment procedure presented in Passarotto and colleagues [31]. Note that *performance quality* is an inverted scale: high scores indicate poor musical performance, and vice versa.

### 2.4. Procedure

Participants took part in the experiment in a quiet room of approximately 9 square meters. At the beginning of the experimental procedure, they provided information about their demographics, musical background, and injury history. The experiment was organized into two main parts: the performance quality assessment and the neuropsychological assessment. 

**Figure 1:**
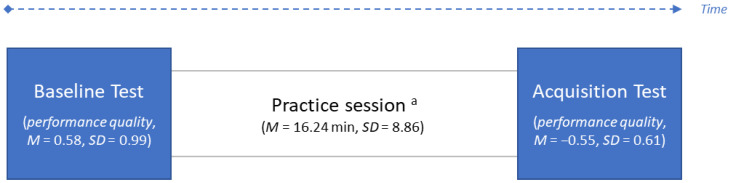
Timeline of the performance quality assessment procedure and descriptive statistics for *time* and *performance quality.* Note: *N* = 30; ^a^ participants were allowed to practice as much as they wanted. The duration of the practice sessions (in parentheses) varied across individuals. *Performance quality* is an inverted scale: high scores indicate poor musical performance, and vice versa.

The performance quality assessment consisted of the same procedure as presented in Passarotto and colleagues [31]. In summary, participants practiced a short musical excerpt inspired by the piano literature on a MIDI keyboard. Participants did not have any previous experience with the musical task or the piece on which this was based. They were free to practice as much as they wanted, using the practice strategies they preferred. As shown in Figure 1, *performance quality* was assessed at baseline and at acquisition, i.e., right before and after the practice sessions. *Time* indicated at what timepoint each performance was recorded, taking individual baseline performances as a reference, when *time* = 0. During each test, musicians were asked to perform the musical excerpt five times, assisted by a metronome. MIDI recordings were used to objectively measure *performance quality* in terms of rhythmic accuracy, wrong and missed pitches, as well as loudness homogeneity through a computerized assessment procedure. For detailed information regarding the performance quality assessment procedure, see Passarotto and colleagues [31].

The neuropsychological assessment consisted of a battery of neuropsychological tasks aimed at investigating executive functions, as detailed above. All tasks were performed on a desktop computer. Participants were seated approximately 50 cm from a monitor with a resolution of 2560 × 1440 pixels and refresh rate of 144 Hz. They completed the tasks via a QWERTZ keyboard and a 1600 dpi corded mouse. The principal investigator (PI) provided the participants with detailed instructions for each task and supervised all introductory practice trials.

The order of the two experimental parts and of the individual NTs within the neuropsychological assessment phase were counterbalanced across participants. The study lasted approximately 2 h. Participants were encouraged to take short breaks at the end of each neuropsychological task and each experimental part. 

### 2.5. Software and Database

The nSTROOP task was developed by the PI according to indications set out by Heine and colleagues [32], using the open-source software OpenSesame [36], version 3.3.11. Open-source versions of computerized DF and TMT tasks by Woods and colleagues [17,18] are publicly available at http://www.ebire.org/hcnlab (accessed in 1 October 2022). The computerized TOL task used in the study is part of the open source PEBL Psychological Test Battery, which can be downloaded at https://pebl.sourceforge.net/battery.html (accessed in 1 October 2022). Statistical analyses were performed in RStudio [37] and through the R-package brms [38]. 

### 2.6. Data Analyses

Between-group t-tests were run to assess differences in NT scores between undergraduate and postgraduate pianists. This allowed us to identify differences in executive functions due to *academic degrees* in music. Correlation matrices were used to investigate the relationship between participants’ *age* and their performance on the NTs.

Multivariate multiple regressions were run to investigate the relationship between *lifetime practice* and NT scores, while accounting for age differences. The model entered the NT parameters as dependent variables and *lifetime practice*, *age* and their interaction (lifetime practice*age) as predictors. 

Bayesian mixed-effects regression models for repeated measures analyses were used to investigate the effect of *time* and NT parameters on *performance quality* scores. The models entered baseline and acquisition *performance quality* scores as criteria, and *time*, *age* and individual NT parameters, as well as their two- and three-way interactions, as fixed effects. *Performer*, with random slopes per *time,* was considered as a random effect. *Time* was coded as 0 for baseline *performance quality* scores, and corresponded to the length of the individual practice sessions for the acquisition *performance quality* scores. Thus, the main effects of the NT parameters and *age* indicated their influence on the model intercepts. The main effect of *time* quantified the participants’ average improvement in *performance quality* per minute of practice. Time*NT parameter interactions described the development of the NT effects during the experiment.

Welch *t*-tests were run to compare participants’ performances on the TMT and DF tasks to the normative data provided by Woods and colleagues [17,18].

## 3. Results

### 3.1. Academic Degrees

The first part of the analyses aimed at identifying differences in NT scores related to *academic degrees* in music. Table 1 reports detailed descriptive statistics for all neuropsychological tasks and task parameters considered in the study, subdivided into *degree* groups. The results showed significant group differences only in TMT scores. Undergraduate students’ performance was significantly better in terms of *tmt_time_b* (completion time of Trail B), *tmt_time_b-a* (difference in completion times between Trail B and Trail A)*, tmt_time_b/a* (ratio between completion times of Trail B and Trail A) and *tmt_errors_b-a* (difference in errors between Trail B and Trail A) scores. Nevertheless, undergraduate students were significantly younger than postgraduate ones, suggesting a possible age-related bias. There were no significant differences in NT scores due to participants’ *gender* (*p* > 0.05). Thus, a post hoc multivariate multiple regression model was run to regress individual NT parameters on *degree*, *age* and degree*age interaction: when accounting for participants’ age, the effect of academic degrees on the NT scores was no longer significant (*p* > 0.05). 

### 3.2. Lifetime Practice

Subsequently, the analyses focused on the relationship between *lifetime practice* and NT parameters. Table 2 reports the results of the multivariate multiple regressions analysis: when accounting for participants’ *age*, *lifetime practice* did not have significant effects on any of the NT parameters, with the only exception being *stroop_rt_size* (the average reaction time in the physical-size comparison part of the nSTROOP task). Thus, high amounts of *lifetime practice* were associated with faster reaction times in evaluating the physical size of visual stimuli. However, this did not generalize to the reaction times measured in the numeric-magnitude comparison part of the nSTROOP task. *Age* was a significant predictor in the model, and it was associated with higher TMT (*tmt_time_b*, *tmt_time_b-a*, *tmt_time_b/a* and *tmt_errors_b-a*) and nSTROOP scores (*stroop_rt_size*).

### 3.3. Performance Quality

**Figure 2:**
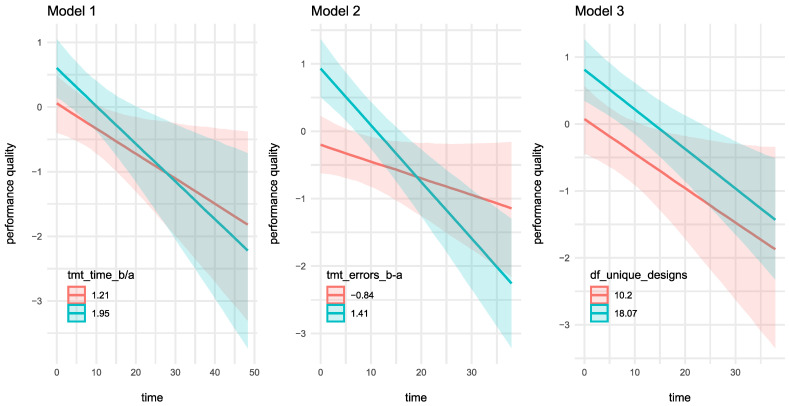
The effect of NT parameters and *time* on *performance quality* scores. Notes: The figure reports model-estimated effects of NT parameters and *time* on *performance quality* scores. For illustration purposes, *performance quality* scores are predicted by considering NT parameters’ values at either one SD below or above the mean of the overall sample. *Performance quality* is an inverted scale (i.e., high scores indicate poor musical performance). Detailed descriptive statistics and effect estimates are reported in Table 1 and Table 3.

The third part of the analyses aimed to investigate the effect of individual NT parameters and *time* on *performance quality* scores, by means of Bayesian mixed effects regression models. *Age* was also entered in the model as a predictor to account for possible age-related differences in NT scores. Table 3 reports the three most relevant models identified during the analyses: *performance quality* scores were predicted by either *tmt_time_b/a* (model 1), *tmt_errors_b-a* (model 2) or *df_unique_designs* (model 3). As shown in Figure 2, *tmt_time_b/a* and *tmt_errors_b-a* had meaningful main effects on *performance quality* scores: large differences in completion times and errors between Trail B and Trail A were associated with poor quality of musical performances. *Df_unique_designs* also had a meaningful effect on *performance quality* scores: a greater number of unique designs drawn in the DF task corresponded to poorer performance in the musical task.

The results showed a meaningful interaction between *time* and *tmt_errors_b-a*, suggesting that the effect of *tmt_errors_b-a* on *performance quality* scores was partially reduced over *time.* No other relevant time*NT interactions were identified. Furthermore, the results showed a significant interaction between *tmt_errors_b-a* and *age*, indicating that the effect of *tmt_errors_b-a* on *performance quality* scores increased with increasing *age*.

### 3.4. Normative Data Comparison

Participants’ performance on the TMT was similar to the normative data provided by Woods and colleagues (2015), with the only exception being the completion times for Trail A (*M* = 25.49 s, *SD* = 5.45) and Trail B (*M* = 39.81 s, *SD* = 10.39), which were significantly faster than the reported reference values (Trail A, *M* = 30.28 s, *SD* = 7.11 s, *t*(82.25) = 3.51, *p* < 0.01 and Trail B, *M* = 46.75 s, *SD* = 16.17, *t*(82.49) = 2.41, *p* < 0.05). Moreover, participants draw significantly more unique designs (*M* = 14.14, *SD* = 3.93) in the DF task than the reported reference values (*M* = 11.98, *SD* = 2.83, *t*(63.04) = 3.14, *p* < 0.01) collected by Woods and colleagues (2016).

Unfortunately, it was not possible to find compatible normative data published (i.e., with similar age and education level) for the nSTROOP and TOL tasks.

## 4. Discussions

The present study investigated the relationship between EFs and musical expertise in university-aged pianists. 

### 4.1. Summary of the Results

The results indicate that postgraduate piano students did not show advantages in EFs compared to undergraduate piano students. More extensive lifetime practice in music was only associated with faster visual reaction times in the physical-size comparison part of the nSTROOP task. TMT and DF scores were significant predictors of the quality of the sample-based musical performance.

In detail, undergraduate students performed better on the TMT performance than postgraduate students. Nevertheless, they were also significantly younger and had cumulated a lower amount of practice during their lifetime. Thus, differences in NT scores were likely due to participants’ age, and not related to academic degrees per se. Subsequent analyses confirmed this hypothesis, showing that when accounting for participants’ age, the effect of academic degrees on the TMT scores was no longer significant. Bayesian mixed effects regression models showed that two parameters from the TMT (*tmt_time_b/a*, *tmt_errors_b-a*) and *df_unique_designs* scores from the DF were related to music performance quality. Large differences in completion times and errors between the Trail B and Trail A of the TMT were associated with poor quality of musical performances. More fluent productivity and a greater number of unique designs drawn in the DF task corresponded to poorer performance in the musical task. The same models identified a meaningful interaction between *tmt_errors_b-a* and *age*, suggesting a possible magnification effect of *tmt_errors_b-a* on music *performance quality* related to participants’ *age*. 

### 4.2. Causal Relationship

The present results indicate that academic degrees in music and the amount of practice cumulated during a lifetime are not meaningful predictors of EFs in advanced learners. Musicians who practiced more and earned higher academic degrees in music performed similarly to their colleagues on the NT tasks, with the only exception being the physical-size comparison part of the nSTROOP task. TMT and DF scores were related to the technical proficiency in the instrument, indicating that specific EFs might provide an advantage in music playing. It could be argued that music playing can promote the development of those EFs that are mostly recruited during music performance and practice. However, the comparison with normative data from other studies does not support this hypothesis, as musicians’ performance in the NTs was not significantly different from the references provided. For these reasons, the present study does not seem to provide evidence for the effects of music training on the development of EFs. 

### 4.3. Inconsistency across Expertise Measures

We investigated the association of EFs and musical expertise across three measures of expertise: academic degree in music, lifetime practice, and performance quality. Most of the meaningful results referred to the technical proficiency in the instrument (*performance quality* scores), evidencing a low level of generalizability of the results across these three measures of musical expertise. The complexity of assessing musical expertise is well known in the literature [30]. As expected, the measures included in this study likely captured different facets of expertise, leading to different results. The same argument might explain the incongruence between our results and the previous literature. For instance, Slevc and colleagues [29] and Okada and Slevc [24] did not find any significant association between musical abilities and set-shifting, but rather with working memory. However, the first study measured expertise in terms of music discrimination abilities, but it did not consider the motor aspect of music playing. The second study used a self-report measurement instrument that assesses participants’ own perception of musicianship, which can be independent of the level of music education attained [5]. Thus, we consider the present findings as complementary to, rather than conflicting with, the literature, as previous studies have considered different facets of musical expertise.

### 4.4. Cognitive Development in Advanced Musicians 

Our results are also in conflict with findings from intervention studies on children and older people [1,25]. One possible explanation may be that the cognitive advantages due to music making do not generalize to high levels of musical expertise, due to ceiling effects in cognitive development. In other words, practicing music might be beneficial to cognitive abilities in the initial stages of learning a musical instrument, with a decreasing effect over time. Alternatively, this effect might depend on learners’ age: music making might be particularly beneficial only in life periods of significant cognitive growth and decline [15,39]. However, to the authors’ knowledge, the literature does not provide any evidence of music interventions on middle-aged adults (i.e., individuals between 20 and 50 years old) and life stages of cognitive stability. Therefore, it is not possible to evaluate the validity of this hypothesis.

### 4.5. Trail Making Task, nSTROOP and Design Fluency 

The results showed that participants’ performance on the TMT and the physical-size comparison part of the nSTROOP task decreased with *age.* These findings are in line with previous studies [16,17] showing age-related increments in completion times for TMT and reaction times for nSTROOP from the third decade of life. 

Participants’ performance on the TMT was similar to the normative data provided by Woods and colleagues [17], with the exception of the completion times for Trail A and Trail B, where musicians were significantly faster than the norm in completing the task but showed similar alternating-switch costs. Moreover, high amounts of practice during a lifetime were associated with faster reaction times in the physical-size comparison part of the nSTROOP task. These findings are in line with the literature showing that adult musicians have a faster auditory and visuospatial processing speed than non-musicians [11,40,41,42]. This is likely promoted by the extensive multisensory integration and temporal processing trained through music practice [43,44], which involve the rapid interpretation of visual stimuli into fine-grained movements.

Lower DF scores were linked to better music performance quality. This might be due to the musical task considered in the experiment, as participants were asked to play the musical excerpt as accurately as possible, performing movements with extreme regularity and little variability. Therefore, the ability to generate multiple geometric patterns while avoiding repetitions was likely detrimental to the objective of the musical task. Nevertheless, participants draw significantly more unique designs in the DF task than the norms provided by Woods and colleagues [18], in line with the literature [11]. Thus, it is important to consider that the present experiment does not represent music practice in its entirety and that design fluency might be beneficial to other aspects of music learning (i.e., expressivity and musicality). 

### 4.6. Limitations

The present study has several limitations. The results might strongly depend on the musical task considered, as different musical activities might rely on different cognitive processes. For instance, creativity might increase the expressivity of a musical performance, which was not measured here. 

The study did not account for possible interindividual differences in socio-economic background or in other variables which might influence executive functioning. Nevertheless, participants had comparable levels of education and musical expertise. Moreover, the study did not include any control group of amateurs or non-musicians, which would have allowed us to make broader inferences regarding participants’ performance in the nSTROOP and TOL tasks. Performance quality was measured only at two timepoints, which was insufficient for measuring the transient effects of individual cognitive abilities: it might be that individual EFs are recruited only at specific intermediate stages of learning a musical piece, rather than after only one practice session. 

### 4.7. Future Developments

Music interventions on healthy, middle-aged adults would help clarify the effect of music training on executive functioning. Moreover, future studies might investigate the long-term effects of suboptimal executive functioning in musicians, specifically assessing whether these are a comorbidity of playing-related injuries.

## 5. Conclusions

In conclusion, the present data show that high amounts of music practice may not be associated with better executive functioning in early adulthood. Nevertheless, some facets of EFs and the quality of musical performance may share substantial amounts of variance, highlighting the importance of optimal cognitive functionality in music practice and performance. 

## Figures and Tables

**Table 1:** NT scores from undergraduate (*N* = 15) and postgraduate (*N* = 20) students, as well as from the complete sample (*N* = 35).

Task	Parameter	Description	Undergraduate	Postgraduate	Overall (*SD*)
TMT	*tmt_time_a*	completion time of Trail A (in seconds)	25.920	25.179	25.497 (5.453)
	*tmt_time_b*	completion time of Trail B (in seconds)	35.471	43.080 *	39.819 (10.394)
	*tmt_time_b-a*	difference in completion times between Trail B and Trail A (in seconds)	9.551	17.901 **	14.323 (8.930)
	*tmt_time_b/a*	ratio between completion times of Trail B and Trail A	1.385	1.731 **	1.583 (0.372)
	*tmt_errors_b-a*	difference in errors ^a^ between Trail B and Trail A	−0.200	0.650 *	0.286 (1.126)
TOL	*tol_moves*	total number of moves across all subtasks	110.067	96.947	102.571 (27.349)
	*tol_time*	completion time of the TOL task (in minutse)	10.228	10.577	10.427 (4.815)
nSTROOP	*stroop_rt_numeric* ^b^	average reaction time in comparing numeric magnitudes (in msec)	515.128	527.381	522.130 (76.631)
	*stroop_rt_size* ^b^	average reaction time in comparing physical sizes (in msec)	373.150	378.501	376.208 (48.114)
	*stroop_delta_rt* ^b^	difference in reaction times between incongruent and congruent conditions (in msec)	38.573	35.941	37.069 (19.396)
	*stroop_delta_err*	difference in errors ^a^ between incongruent and congruent conditions	1.733	1.501	1.600 (1.418)
DF	*df_unique_designs*	number of unique designs drawn	15.067	13.45	14.142 (3.934)
	*df_repeated_abs*	number of designs which were drawn more than once	0.933	0.801	0.857 (1.331)
	*df_repeated_pct*	ratio of repeated trials to the overall number of designs	0.050	0.046	0.047 (0.068)
	*Age*	participants’ age (in years)	21.60	26.15 ***	24.20 (3.826)
	*Years of practice*	years of music learning	14.67	19.40 ***	17.37 (3.89)
	*Lifetime practice*	cumulative amount of practice hours during lifetime	13,093	20,451 *	17,298 (9934)

Notes: *N =* 35; * between groups *t*-test significant at *p* < 0.05, ** between groups *t*-test significant at *p* < 0.01, *** between groups *t*-test significant at *p* < 0.001, ^a^ number of wrong responses, ^b^ measured on correct trials only. TMT = Trail Making Task, TOL = Tower of London, nSTROOP = numerical STROOP, DF = Design Fluency. When accounting for participants’ *age*, the results did not indicate any significant difference in NT scores related to *academic degrees* (*p* > 0.05).

**Table 2:** The effect of age and lifetime practice on NT scores.

Task	DV	Intercept	Age	Lifetime Practice	Age: Lifetime Practice
TMT	*tmt_time_a*	0.086	−0.057	−0.089	−0.332
	*tmt_time_b*	0.064	0.395 *	−0.230	−0.247
	*tmt_time_b-a*	0.069	0.380 *	−0.162	−0.265
	*tmt_time_b/a*	−0.021	0.475 *	−0.153	0.080
	*tmt_errors_b-a*	0.022	0.494 **	−0.213	−0.085
TOL	*tol_moves*	0.049	−0.301	−0.022	−0.187
	*tol_time*	0.027	−0.201	0.274	−0.104
nSTROOP	*stroop_rt_numeric* ^a^	0.032	0.258	−0.247	−0.124
	*stroop_rt_size* ^a^	0.160	0.337 *	−0.358 *	−0.617 *
	*stroop_delta_rt* ^a^	0.021	0.027	−0.093	−0.079
	*stroop_delta_err*	0.023	−0.240	0.233	−0.090
DF	*df_unique_designs*	−0.095	0.114	−0.169	0.366
	*df_repeated_abs*	0.068	0.094	−0.177	−0.261
	*df_repeated_pct*	0.097	0.130	−0.185	−0.372

Notes: *N* = 35; * beta coefficient is significant at *p* < 0.05, ** beta coefficient is significant at *p* < 0.01, ^a^ reaction time measurements have been log-transformed to compensate for their skewness. TMT = Trail Making Task, TOL = Tower of London, nSTROOP = numerical STROOP, DF = Design Fluency. For the analyses, all dependent variables and predictors have been standardized across participants. For a description of each task parameter, see Table 1.

**Table 3:** The effect of NT parameters and *time* on *performance quality* scores.

	Model 1	Model 2	Model 3
Fixed Effects	Estimate [95% CI]	Estimate [95% CI]	Estimate [95% CI]
Intercept	0.36 [0.06, 0.65]	0.37 [0.07, 0.66]	0.37 [0.07, 0.66]
*time*	−0.05 [−0.07, −0.03]	−0.05 [−0.08, −0.03]	−0.05 [−0.08, −0.03]
*age*	−0.06 [−0.39, 0.28]	−0.01 [−0.30, 0.26]	−0.01 [−0.30, 0.26]
*time: age*	0.00 [−0.02, 0.03]	0.01 [−0.01, 0.03]	0.01 [−0.01, 0.03]
*tmt_time_b/a*	0.30 [0.00, 0.62]	-	-
*time: tmt_time_b/a*	−0.01 [−0.04, 0.02]	-	-
*age: tmt_time_b/a*	0.14 [−0.11, 0.40]	-	-
*time: age: tmt_time_b/a*	0.01 [−0.01, 0.03]	-	-
*tmt_errors_b-a*	-	0.57 [0.26, 0.87]	-
*time: tmt_errors_b-a*	-	−0.03 [−0.05, −0.01]	-
*age: tmt_errors_b-a*	-	0.38 [0.00, 0.78]	-
*time: age: tmt_errors_b-a*	-	0.01 [−0.02, 0.04]	-
*df_unique_designs*	-	-	0.35 [0.04, 0.68]
*time: df_unique_designs*	-	-	−0.01 [−0.03, 0.01]
*age: df_unique_designs*	-	-	−0.01 [−0.36, 0.34]
*time: age: df_unique_designs*	-	-	0.00 [−0.02, 0.03]
Random Effects			
Performer:			
Intercept	0.41 [0.23, 0.59]	0.41 [0.24, 0.58]	0.41 [0.24, 0.59]
*time*	0.01 [0.00, 0.03]	0.01 [0.00, 0.03]	0.02 [0.00, 0.03]
cor (Intercept, *time*) ^a^	−0.17 [−0.79, 0.60]	−0.10 [−0.76, 0.65]	−0.11 [−0.75, 0.62]
residuals	0.76 [0.59, 0.96]	0.69 [0.52, 0.88]	0.78 [0.62, 0.98]
Coefficients of determination			
Conditional R^2^	0.54 [0.39, 0.69]	0.60 [0.45, 0.73]	0.48 [0.33, 0.63]
Marginal R^2^	0.45 [0.31, 0.56]	0.48 [0.36, 0.59]	0.39 [0.25, 0.50]

Notes: *N* = 30; ^a^ correlation between random effects. *Performance quality* was predicted by either *tmt_time_b/a* (model 1), *tmt_errors_b-a* (model 2) or *df_unique_designs* (model 3). *Performance quality* is an inverted scale (i.e., high scores indicate poor musical performance). *Age*, *tmt_time_b/a*, *tmt_errors_b-a* and *df_unique_designs* were standardized across participants. The table reports fixed and random effects, followed with 95% credible intervals in square brackets [ ]. For a description of each NT parameter, see Table 1.

## Data Availability

The database and the R script used in the study are publicly available at the following link: https://osf.io/u2bgt (accessed on 29 May 2023).
